# Nutrient Supplementation during the Prenatal Period in Substance-Using Mothers: A Narrative Review of the Effects on Offspring Development

**DOI:** 10.3390/nu15132990

**Published:** 2023-06-30

**Authors:** Catherine A. Serwatka, Adrianne K. Griebel-Thompson, Rina D. Eiden, Kai Ling Kong

**Affiliations:** 1 Baby Health Behavior Laboratory, Division of Health Services and Outcomes Research, Children’s Mercy Research Institute, Children’s Mercy Hospital, Kansas City, MO 64108, USA; cserwatka@cmh.edu (C.A.S.); adrianne.griebel@gmail.com (A.K.G.-T.); 2 Department of Psychology and the Social Science Research Institute, The Pennsylvania State University, University Park, PA 16801, USA; rina.eiden@psu.edu; 3 Department of Pediatrics, University of Missouri-Kansas City, Kansas City, MO 64110, USA; 4 Center for Children’s Healthy Lifestyles and Nutrition, University of Kansas Medical Center, Kansas City, KS 66160, USA

**Keywords:** pregnancy, substance use, nutrient supplementation, development

## Abstract

Substance use during pregnancy increases the risk for poor developmental outcomes of the offspring, and for substance-dependent mothers, abstaining from substance use during pregnancy is often difficult. Given the addictive nature of many substances, strategies that may mitigate the harmful effects of prenatal substance exposure are important. Prenatal nutrient supplementation is an emerging intervention that may improve developmental outcomes among substance-exposed offspring. We provide a narrative review of the literature on micronutrient and fatty acid supplementation during pregnancies exposed to substance use in relation to offspring developmental outcomes. We first discuss animal models exposed to ethanol during pregnancy with supplementation of choline, zinc, vitamin E, iron, and fatty acids. We follow with human studies of both alcohol- and nicotine-exposed pregnancies with supplementation of choline and vitamin C, respectively. We identified only 26 animal studies on ethanol and 6 human studies on alcohol and nicotine that supplemented nutrients during pregnancy and reported offspring developmental outcomes. There were no studies that examined nutrient supplementation during pregnancies exposed to cannabis, illicit substances, or polysubstance use. Implementations and future directions are discussed.

## 1. Introduction

Prenatal substance use is a public health concern. There have been significant variations in the prevalence of different substances used during pregnancy across the last three decades [1]. Contemporary data indicate that among pregnant women 18–49 years of age, 13.5% consume alcohol, and 5.2% reported binge drinking, which is defined as having four or more drinks on a single occasion within the last month [2]. Further, 8.4% of pregnant women reported tobacco use, 0.4% used illicit drugs other than cannabis, and 8.0% used cannabis within the last month [3]. There is an extensive body of research indicating an increased risk for mother–infant morbidity and poorer developmental outcomes across time as a function of prenatal substance exposure. For instance, chronic heavy alcohol exposure is associated with an increased risk for compromises in orientation and motor maturity [4], growth deficits [5], facial anomalies [6], reductions in IQ [7], central nervous system malfunction [8], and damage to the hippocampus and corpus collosum [9]. Together, these problems are often referred to as fetal alcohol spectrum disorders (FASDs), which are classified as neurobehavioral disabilities [10]. Meanwhile, prenatal tobacco exposure, in the form of combustible cigarettes, is associated with an increased risk for poor developmental outcomes such as low birthweight [11], reduced brain size [12], and altered brain function [13]. Tobacco exposure is also associated with an increased risk of obesity [14], hypertension [15], asthma [16], and wheezing [17] later in life. Finally, cannabis, which is an increasingly popular drug of choice among pregnant mothers due to its perceived safety and widespread legalization [18], has been linked to reductions in offspring birthweight and birth length [19], learning disabilities, and memory impairments [20,21,22].

Traditionally, healthcare officials called for the abstinence from the aforementioned substances to promote growth and development and to prevent risk to the mother and fetus. However, this approach is problematic for several reasons. First, nearly half (45%) of all pregnancies are unintended [23]; therefore, prenatal substance exposure may occur before women realize they are pregnant. This results in fetal exposure during a critical period of brain development and may increase the risk of negative outcomes across the lifespan of the fetus [24]. Second, although there are dramatic reductions in the amount of use upon pregnancy recognition, pregnant individuals with heavier patterns of use continue to use substances [25]. Given the difficulties associated with quitting or abstaining from substance use for those with heavier use patterns, alternate harm reduction strategies that promote fetal health may be useful. One small prospective study (*n* = 152) on pregnant women with heavier use patterns revealed that 4% of women who are classified as ‘heavy drinkers’ (i.e., having an average of ≥1.0 oz of absolute alcohol per day or ≥4 or more standard drinks per occasion), 22% of pregnant individuals who use cannabis, 27% of pregnant individuals who snort cocaine, and 68% of individuals who smoke cigarettes on a regular basis continued to engage in these behaviors after learning of their pregnancy [26].

There is an accumulating interest in using prenatal nutrient supplementation as a strategy that may reduce the risk for poorer fetal outcomes after prenatal substance exposure. Vitamins and minerals are vital for optimal fetal development. For instance, choline aids cell division and lipid transport [27]. Zinc, the second most abundant metal in the body, is integral to central nervous system development through myelination and gene expression [28]. Iron is an essential nutrient during pregnancy, and the need for iron increases in order to aid cellular functions in delivering oxygen to support the structural development of fetal organs [29]. Vitamin E regulates embryonic development and placental maturation while also protecting the fetus from oxidative stress [30]. Finally, vitamin C is essential to cognitive development by way of normative hippocampal growth [31]. The supplementation of macronutrients such as omega-3 fatty acids (i.e., docosahexaenoic acid (DHA)) during pregnancy was shown to improve developmental outcomes including better visual acuity and performance on the Mental Development Index in children up to 4 years of age [32]. Accordingly, it is plausible that increased intakes of micronutrients and/or fatty acids amid the prenatal period may help to offset or mitigate the adverse effects of the in utero exposure to substance use on infant development.

For this narrative review, we synthesized the existing literature on nutrient supplementation in pregnant women using substances, focusing on how supplementation influences physical, cognitive, and behavioral development in animals and humans. Upon data extraction, we were unable to locate empirical studies that included substances other than alcohol/ethanol and nicotine. Thus, the following review will be solely focused on nutrient supplementation during the prenatal period in pregnancies with alcohol and nicotine exposure and offspring outcomes.

## 2. Materials and Methods

### 2.1. Search Strategy

We conducted a full electronic search within PubMed in January 2023. Search terms encompassed variations of “prenatal substance use”, “maternal nutrition”, “supplementation”, “offspring outcomes”, and “development outcomes” (see Appendix A for full search strategy). Potential papers were screened by title and abstract.

### 2.2. Eligibility Criteria

Studies were eligible for inclusion if they (1) were published in an academic, peer-reviewed journal and written in English, (2) investigated prenatal nutrient supplementation and substance exposure in animals or humans, and (3) reported on developmental outcomes in the offspring. Studies were excluded if (1) pregnant individuals did not receive nutrient supplements or use substances during the prenatal period, and (2) outcomes reported were unrelated to offspring development. All studies were imported into EndNote 20.0 software for review.

### 2.3. Data Extraction

**Figure 1:**
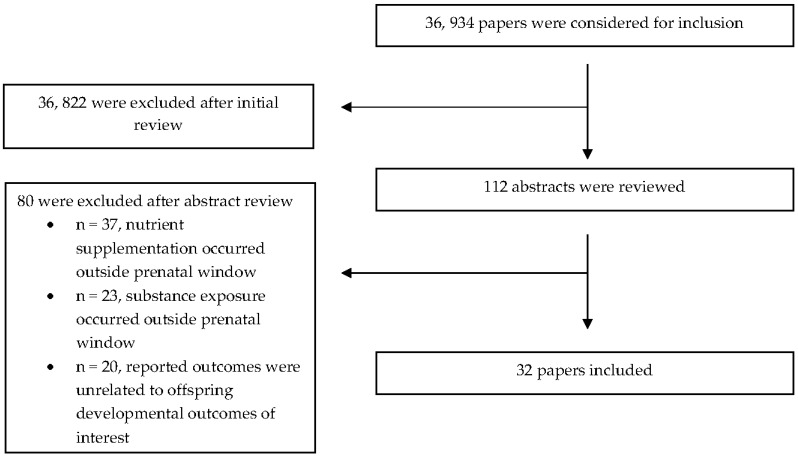
Flow chart of excluded and included studies.

After duplicate removal, we assessed 36,934 studies. Of these studies, 112 had abstracts that warranted a full text review (Figure 1). Two separate investigators (C.S. and A.K.G.T.) evaluated whether the (1) design, (2) timing and duration of nutrient supplementation, and (3) outcomes reported were relevant to the topic at hand. A third investigator (K.K.) further reviewed these studies in order to find a consensus over which ones met the inclusion criteria for the present study. Following this discussion, we decided to exclude 80 studies because they did not meet inclusion criteria. In total, 32 studies are present in this narrative review, and a full summary of their characteristics, methodology, and findings, organized alphabetically by first author’s last name, can be found in Table 1 and Table 2.

## 3. Results

### 3.1. General Study Characteristics

Table 1 and Table 2 provide a description of the general study characteristics and findings. We reported on 32 studies investigating the interactive effects of nutrient supplementation and substance use during the prenatal period on developmental outcomes in offspring. Of these studies, 26 were experimental studies in animals (16 were conducted on rats, 7 on mice, 3 on ewes), and 6 were observational studies in humans. In the animal studies, we included rodent studies that supplemented nutrients on postnatal days (PDs) 1–10, because these days are the equivalent to the human third trimester. The rodent gestation is accelerated in comparison to human gestation, with the first trimester equivalent to gestational days (GDs) 1–10, the second trimester equivalent to GDs 11–20, and the third trimester and cognitive brain growth spurt corresponding to PDs 1–10 [68].

### 3.2. Animal Studies

All animal studies solely investigated the effects of maternal nutrient supplementation on prenatal ethanol exposure. The breakdown of the following outline is based on micro- (i.e., choline, zinc, vitamin E, and iron) and macronutrient supplementation (i.e., fatty acids) under the backdrop of ethanol exposure in animal studies.

#### 3.2.1. Effects of Choline on Maternal Ethanol Consumption and Offspring Developmental Outcomes

Choline was the predominant micronutrient investigated, as it was examined in 18 studies. More than half of these studies (11/18) found varying positive effects of choline on the outcomes of interest [34,36,40,41,43,44,45,47,48,49,50]. From the studies that found positive protective factors from choline supplementation against ethanol, five reported an ameliorating effect of choline on preventing the reduction in body, brain, liver, and heart weights, and the brain-to-liver weight ratio, as well as an increased length of gestation (an indicator of increased fetal body weight) [36,40,44,48,68]. Three studies reported choline’s positive effect on motor coordination and balance [34,36,44], and three more found a positive effect on working memory and learning [47,49,50]. According to two studies, choline contributed to significant reductions in hyperactivity levels [41,47], and another found that choline improved visuospatial discrimination acquisition [45]. Finally, choline had positive effects on craniofacial abnormalities [43] and was found to partially ameliorate anxiety-like behaviors [36]. It is important to note the study conducted by Thomas et al. [49], which investigated the concurrent administration of ethanol with choline during the prenatal period. It found mitigating effects of choline on body weight growth alongside behavioral tasks including spontaneous alteration and spatial working memory. Specifically, the ethanol-exposed subjects who were treated with choline performed at levels that did not significantly differ from the performance levels of the control group that was not exposed to ethanol on the tasks described [49].

In contrast, six of the studies found that choline had no effects on growth [33,35,37,38,39,42], and one found no positive effects of choline on motor coordination [46]. Among the outcomes that did not have any protective effects associated with choline supplementation included whole brain measurements, cognition, and attention [35,39]. Table 1 summarizes these studies.

#### 3.2.2. Effects of Zinc on Maternal Ethanol Consumption and Offspring Developmental Outcomes

Three animal studies by Summers et al. [51,52,53] investigated zinc supplementation against ethanol exposure, and all three reported positive effects. Table 1 displays these findings. These studies used the same condition in which mice were injected with 25% ethanol on gestational day 8 (i.e., first trimester equivalent) or a saline control. Zinc supplementation was administrated on gestational day 8 or throughout both the first and second trimester equivalent (i.e., gestational days 1–18) [52,53]. Summers et al. [51] investigated the cognitive impairments of offspring after maternal ethanol exposure and found that zinc supplementation helped to improve cognitive outcomes. The offspring of mothers who were exposed to ethanol and zinc during pregnancy performed at the same level as the offspring of mothers who were exposed to saline control for the cross-maze water escape task, which measures spatial learning and recall in memory. This work was replicated, and the findings indicated that the offspring of the ethanol plus zinc group performed at the same level as the saline control group on the cross-maze water escape and object recognition memory tasks [52]. Finally, Summers at al. (2009) reported that zinc supplementation protected against incidences of physical abnormalities and postnatal mortality. In the ethanol-exposed group without zinc treatment, the authors observed a 35% occurrence for both physical abnormalities and postnatal mortality. When zinc was supplemented, only 12% of the offspring from mothers who were exposed to ethanol had physical abnormalities. This is a relatively comparable risk to the 10% in the saline group and the 9% in the saline plus zinc group. Similarly, when zinc was supplemented, postnatal mortality was only 12%, which is lower than both the saline group (14%) and saline plus zinc group (18%) [53].

#### 3.2.3. Effects of Vitamin E on Maternal Ethanol Consumption and Offspring Developmental Outcomes

Two animal studies investigated the protective effects of vitamin E against prenatal ethanol administration during the third trimester equivalent in humans (i.e., PD 6 and PDs 4–9), and neither study found protective effects [54,55]. Marino et al. [54] supplemented vitamin E on postnatal days 6–9, prior to ethanol exposure on days 7–9, and did not find any mitigating effects on the reductions in body weight; fetuses born to dams in the ethanol only and ethanol plus vitamin E groups had significantly lower body weights on postnatal days 8 and 9 compared to the control group. Furthermore, the Morris water maze task given to the ethanol-exposed subjects indicated that vitamin E supplementation did not improve impairments in spatial navigation [54]. In the study conducted by Tran et al. [55], vitamin E supplementation occurred concurrently with ethanol exposure on postnatal days 4–9 and failed to protect against deficits in functional learning outcomes, which were measured using eyeblink conditioning [55].

#### 3.2.4. Effects of Fatty Acids on Maternal Ethanol Consumption and Offspring Developmental Outcomes

Two animal studies supplemented gamma-linolenic acid [57] and saturated fats versus polyunsaturated fats [56] against the teratogenic effects of ethanol and reported mixed findings. The first study, conducted by Wainwright et al. [57], investigated the supplementation of gamma-linolenic acid, a long-chain fatty acid, during pregnancy on acute ethanol exposure. The supplementation occurred concurrently with ethanol exposure from gestational days 7 to 17 (i.e., first and second trimester equivalent). They did not find attenuating effects of gamma-linolenic acid on reductions in body weight and brain weight. Additionally, there were no effects on any of the behavioral outcomes of interest, such as a battery of tests measuring reflex response, open field behaviors, and locomotor activity. The second study, conducted by Abel and Reddy [56], investigated the effects of a high saturated fat diet compared to a high polyunsaturated fat diet against teratogenic ethanol exposure on hyperactivity levels in rats, which is a common behavioral characteristic of children born with FASD [10]. The alcohol-exposed offspring who were fed a diet high in saturated fat had lower hyperactivity levels than their pair-fed controls, and these offspring also engaged in less anxiety-like behaviors such as head-dipping. In contrast, the high polyunsaturated fat diet did not significantly improve alcohol’s effects on hyperactivity and anxiety-like behaviors [56].

#### 3.2.5. Effects of Iron on Maternal Ethanol Consumption and Offspring Developmental Outcomes

The one remaining animal study supplemented elemental iron [58] against the embryotoxic effects of ethanol and reported one positive finding relating to physical development. Helfrich et al. [58] explored the supplementation of iron during pregnancy on acute ethanol exposure, with concurrent ethanol exposure and iron supplementation occurring from gestational days 13.5 to 19.5 (i.e., second trimester equivalent). They found significant mitigating effects of iron supplementation on absolute brain weight in male pups exposed to ethanol, but not females. There were no other interactive effects of iron supplementation against ethanol exposure for birth weight or liver and heart weights in either male or female pups.

### 3.3. Human Studies

Studies involving human participants investigated the effects of choline on prenatal alcohol consumption (*n* = 4 studies) [59,60,61,63] and the effects of vitamin C on nicotine exposure (*n* = 2 studies) [64,65].

#### 3.3.1. Effects of Choline on Maternal Alcohol Consumption and Offspring Developmental Outcomes

Four studies examined the potential protective effects of choline supplementation against alcohol exposure either alone [60,63] or in combination with a multivitamin [59,61]. The results were mixed. Two studies [60,63] found positive protective effects of choline on alcohol-exposed pregnancies. Jacobson et al. [60] recruited heavy drinkers, which they defined as having an average consumption of at least two standard drinks (1.0 oz absolute alcohol) per day or at least one incident of binge drinking (four or more standard drinks per occasion) over the course of pregnancy. They were randomly assigned to either the placebo group or 2 g choline supplementation group at the 23rd week of gestation. The choline supplementation demonstrated protective effects for physical and cognitive development; the choline-treated infants showed considerable catch-up growth in weight and head circumference at 6.5 and 12 months. These infants were more likely to meet the criterion for eyeblink conditioning and showed greater increases in conditioned responses across the three training sessions. At 12 months, the infants in the choline treatment arm had better visual recognition memory as measured by the Fagan Test of Infant Intelligence in comparison to the controls who were exposed to ethanol but did not receive choline supplementation [60]. Warton et al. [63] recruited heavy drinkers, which they defined as having an average consumption of two standard drinks (1.0 oz absolute alcohol) per day or at least one incident of binge drinking (four or more standard drinks per occasion) during pregnancy, who were enrolled at 23 weeks’ gestation. When 2 g of choline per day was supplemented to the women, the authors observed an improvement in brain structural outcomes of the offspring. Specifically, infants exposed to choline showed larger brain volumes in 6 of the 12 regions. The development of the larger right putamen and corpus callosum regions were associated with higher scores on the Fagan Test of Infant Intelligence [63].

In contrast to the first two studies, the two studies by Coles et al. [59] and Kable et al. [61], and the follow-up findings from the original randomized controlled trial (RCT) by Kable [62], paired choline with a multivitamin and found that this showed no effects on the offspring of heavy drinkers. Coles et al. [59] recruited heavy drinkers, which they defined as (1) having at least weekly binge episodes (5 + drinks), (2) at least five episodes of 3–4 standard drinks, or (3) at least ten episodes of 1–2 standard drinks either in the month around conception or the most recent month of pregnancy. The participants were assigned to one of the following three groups: (1) regular diet with a recommendation to take a multivitamin but were not provided with one, (2) regular diet with a multivitamin provided, or (3) regular diet with a multivitamin and 750 mg of choline supplement provided. Among the offspring of women who were assigned to the multivitamin and choline supplements group, the author did not observe improved scores on the Psychomotor Development Index and Mental Development Index. Additionally, the behavioral rating in the Bayley Scales of Infant Development 2nd Edition did not indicate improvement in areas of orientation/engagement, emotional reactivity, motor quality, or total behavior. Interestingly, the Mental Development Index showed that the multivitamin only group scored significantly higher than the multivitamin plus choline group [59].

In the original RCT reported by Kable et al. [61], heavy drinkers were assigned to one of the following three groups: (1) mother’s regular diet with a recommendation to take a multivitamin supplement that is not provided by the study, (2) mother’s regular diet plus a provided multivitamin supplement, and (3) regular diet plus a provided multivitamin and 750 mg of choline supplement. This study reported no mitigating effects of multivitamin and choline supplements on reductions in birth weight, length, or head circumference in alcohol-exposed infants. Furthermore, choline did not significantly affect cardiac-orienting responses to the auditory stimuli, nor did it significantly affect latency responses in the visual habituation tasks, which are measures of cognitive development. However, the prenatal supplementation of multivitamins and choline resulted in a significantly greater change in the heart rate in the visual habituation task, which is an index of early cognitive functioning, in children between 6–12 months of age in both the ethanol-exposed and control groups [61]. Finally, Kable et al. [62] reported follow-up results from an RCT on the reaction time of 4-year-old preschoolers (mean age 3.96 years). These preschoolers were participants from a previous study conducted in 2015 [61]. The authors reported that although multivitamin supplementation improved the reaction time performance in male preschoolers, it did not have the same effect on female preschoolers. Additionally, choline supplementation improved the reaction time in male preschoolers who were not exposed to alcohol, but not in those who were exposed to alcohol or in female preschoolers [62]. This was the only study in which the pairing of choline supplementation with a multivitamin, which contains a variety of vitamins and minerals, produced positive effects.

#### 3.3.2. Effects of Vitamin C on Maternal Nicotine Consumption and Offspring Developmental Outcomes

Two studies [64,65] and two follow-up studies [66,67] investigated the ameliorating effects of vitamin C against nicotine exposure, and all showed positive effects of vitamin C supplementation on physical development, specifically the pulmonary function of infants, both at the original timepoint, as well as at the follow-up timepoints. Across both studies, current smokers who smoke at least one cigarette per day were randomized to the vitamin C supplementation group or to the placebo pill group at ≤22 weeks’ gestation. In a stand-alone study by McEvoy et al. [64], pulmonary assessments completed at 3 days of age and 12 months of age reported effects in offspring. In addition to the stand-alone study, McEvoy et al. [65] are conducting an ongoing RCT [65], which follows children from 3 months of age at the original timepoint to 12 months of age [66] and 5 years of age [67] in the respective follow-up timepoints. Despite the variance in the assessment age, the results of both studies largely show positive effects of vitamin C supplementation on pulmonary outcomes. In the first stand-alone study, McEvoy et al. [64] reported that vitamin C supplementation had a positive effect on incidences of wheezing. In assessments of infants from three days though one year of age, the infants of women who received vitamin C had significantly decreased incidences of wheezing in comparison to those who received the placebo. The participants in the ongoing RCT [65] with follow-ups through 5 years of age differ from the previously reported 2014 stand-alone study, as they are a part of the report on the use of vitamin C to decrease the effects of smoking in pregnancy on infant lung function (VCSIP) cohort. The study by McEvoy et al. [65] was the first to report VCSIP findings, and it found additional positive effects. Infants whose mothers were randomized to receive vitamin C during pregnancy had an increased forced expiratory flow at 75% of the expired volume during a pulmonary function test as well as a significantly increased forced expiratory flow at 50% of the expired volume at 3 months of age compared with those who were randomized to receive the placebo [65]. In the next report of VCSIP findings, McEvoy et al. [66] reported similar positive results. Forced expiratory flows in the vitamin C-treated group increased from 11.6% to 16.1% in comparison to the placebo group, and vitamin C produced a persistently significant increase in the offspring’s airway function at 3 and 12 months of age [66]. The final VCSIP study, McEvoy et al. [67], is the most recent follow-up study, and it reports that supplemental vitamin C contributes to significantly better airway function and a lower occurrence of wheezing in offspring at 5 years of age.

## 4. Discussion

Our review of the literature finds that the implementation of nutrient supplementation to ameliorate child risk for negative outcomes related to prenatal substance exposure is promising. Although preclinical models support the finding that micronutrient zinc improves the developmental outcomes of ethanol-exposed fetuses during pregnancy, this intervention has yet to be examined in studies with human participants. Additional studies in preclinical models provide less evidence, though positive results, for the use of choline, iron, and fatty acids, but none of the studies regarding vitamin E found that supplementation improved offspring developmental outcomes. As for studies involving human participants, only choline and vitamin C supplementation have been investigated regarding alcohol and nicotine exposure, respectively. Similar to the preclinical models, these studies found that the efficacy of choline supplementation after alcohol exposure is mixed, though mostly positive. Other studies, such as those examining vitamin C, suggest that its supplementation improves pulmonary outcomes in fetuses with nicotine exposure. However, the included studies varied greatly regarding the timing of nutrient supplementation and the timing and dose of ethanol exposure, assessed developmental outcomes, study design, and prenatal nutrient of interest. Taken together, ethanol exposure during the prenatal period increases the risk for cognitive, behavioral, and neural changes in the fetus. These alterations vary based on the dosing, duration, and timing of the ethanol exposure. Moreover, substances may alter fetal development through a variety of possible mechanisms; ethanol exposure during pregnancy induces cell and mitochondrial damage, which leads to central nervous system dysfunction [69]. Additionally, fetal ethanol exposure produces alterations in DNA methylation and microRNA expression and function in zebra fish, leading to developmental deficits [70]. Furthermore, nicotine is an addictive substance that readily crosses the placenta, and the fetal concentrations of the compounds are higher than the maternal concentrations alone. This increases the risk for a number of outcomes such as poor regulation of arousal within the first month of life to cognitive deficits, including language comprehension and sensory processing, persisting through 18 years of age [24]. It is plausible that supplementations of nutrients such as zinc, vitamin C, iron, and choline may work to ameliorate these poor developmental outcomes as they play essential roles in growth, DNA synthesis, and neurodevelopment. For instance, possible mechanisms of choline’s effects in FASD include its role in the production of phospholipids that are essential for growth and myelination [71]. Additionally, choline supplementation also affects choline acetyltransferase levels in the hippocampus in animal models, resulting in improved memory [72]. Moreover, vitamin C may reduce ethanol’s effects on oxidative stress because it stops the production of peroxidative stress with the help of vitamin E [73]. Notably, iron is essential to the uptake of oxygen needed by the developing fetal brain, which accounts for 60% of the fetal oxygen consumption rate [74]. Finally, zinc plays an essential role in a variety of complex mechanisms including cell division and replication, gene expression, and hormone regulation [75].

The substances administered (i.e., ethanol in animal studies and alcohol and nicotine in human studies) are also limited, as none of these studies examined substances such as cannabis, prescription pain medications, or illicit opioids, nor did they examine polysubstance use, despite this being a common pattern of use among heavier users or users of illicit substances including opioids. However, half (3/6) of the human samples [60,61,63] included polysubstance use during pregnancy among samples recruited for alcohol or nicotine use. Specifically, Jacobsen et al. [60] reported high levels of cigarette use (1/4 pack/day), and four participants reported the use of methamphetamine later in pregnancy in addition to heavy prenatal alcohol consumption. Moreover, Kable et al. [61] and Warton et al. [63] also reported that women who consumed alcohol while pregnant had significantly higher cigarette use, and Warton et al. [63] observed higher cannabis use in the alcohol-using women as well. As a result, this review summarizes the efficacy of prenatal micronutrient supplementation as a potential protective factor for fetuses on ethanol-/alcohol- and nicotine-exposed pregnancies.

While this review shows promise for the ability of micronutrient and fatty acid supplementation to protect developing fetuses from harmful substances based on animal models, our search of the literature reveals that there are few studies involving human participants. One explanation for the lack of human studies may be due to the general stigma surrounding prenatal substance exposure and the subsequent hesitancy of expectant mothers to report their use accurately and honestly due to the fear of losing custody of their child [76]. Garg et al. [77] reported that substance use during pregnancy is substantially underreported for commonly consumed substances, even among women who regularly participate in urine substance screens [77]. This suggests that the number of pregnant women actively engaging in illicit substance use may be even higher than the current reported rate. Additionally, pregnant individuals with substance use disorder face many relational and structural barriers, including perceived judgmental attitudes of health care providers and a lack of access to treatment for lower income women and women of color [77]. Though not a commonly reported reason, according to a study conducted by Borland and colleagues, women choosing to continue the use of substances during pregnancy could be due to the perceived fear of causing stress and undue harm to the fetus as a result of quitting substance use [78]. For example, exposing the fetus to increased levels of psychological stress as a result of quitting was perceived by some women to be more harmful for the baby than the substance use itself. Thus, nutrient supplementation may play a vital role in providing protection to fetuses of pregnant women who employ harm reduction strategies, rather than abstinence, while pregnant.

As preclinical and translational research continues to unfold, the next step to protect fetuses from substance-use-related harm during pregnancy is to begin implementing these important findings. To achieve this, we suggest the following: first, while outside of the scope of this review, ensuring access to quality prenatal care beginning in the first trimester is essential to improve fetal outcomes for pregnant individuals with substance use disorders. Improved levels of prenatal care are associated with a reduced risk of poor pregnancy outcomes after substance use [79]; however, pregnant individuals with substance use disorders face many barriers to receiving adequate prenatal care [80]. It is pivotal to develop strategies that eliminate these barriers, allowing for this population to have an improved access to prenatal care. Particularly, many prenatal healthcare providers are the sole suppliers of supplements to pregnant individuals who may not possess the resources to obtain these supplements on their own. Therefore, regular visits to a prenatal healthcare provider offers an avenue for free supplementation that can be beneficial to the development of the fetus. Next, the consumption of a diet that is rich in meats (i.e., zinc, choline, saturated fats, iron), eggs (i.e., choline, long-chain fatty acids), fatty fish (i.e., long-chain fatty acids), and fruits and vegetables (i.e., vitamin C, iron) is recommended in order to obtain the nutrients that were shown to protect fetuses from harmful substances. Encouraging eligible women to enroll in programs like the Special Supplemental Nutrition Program for Women, Infants, and Children (WIC) and the Supplemental Nutrition Assistance Program (SNAP) is a strategy that improves food and nutrition security in households where the access to nutritious food is limited. Last, the consumption of a high-quality prenatal supplement is recommended. This supplement must contain adequate levels of choline and long-chain fatty acids (i.e., DHA), which are two nutrients that are commonly found in inadequate amounts in both prenatal supplements and the typical American diet. Perhaps future policies and procedures can center on improving the intake of high-quality prenatal supplements for pregnant individuals with substance use disorders by making these prenatal supplements accessible through their providers.

There are limitations to this review. First, the literature regarding prenatal supplementation is limited in terms of the quantity and heterogeneity in its methodological designs. Of the studies which investigated prenatal alcohol consumption, none intentionally investigated the mitigating effects of micronutrient supplementation on polysubstance use, despite the recent reports that concurrent substance use is as high as 40% in pregnant women who consume alcohol [81]. Additionally, the small sample size of the included studies is a limitation. Thus, future review papers or meta-analyses on this topic could consider expanding their search terms to encompass a wider range of nutrient supplementation. Furthermore, no study investigated the effects of nutrient supplementation on illicit substance exposure, despite our knowledge that illicit substance use during pregnancy can produce deleterious effects on pregnancy and fetal outcomes. Another limitation is the substantial differences in the methodological approaches across animal studies. For instance, there appeared to be no consensus on the timing of micronutrient supplementation and the administration of ethanol, or there are large variations in ethanol dose during pregnancy. Although a slim majority reported both micronutrient supplementation and ethanol use during the prenatal period, the specific prenatal days in which the nutrient supplementation was provided and the occurrence of ethanol exposure varied greatly. In addition, all animal studies reported the use of ethanol-exposed models without further study of prenatal substance exposure models beyond this area. The lack of human studies serves as a limitation as rodents have an accelerated gestation, and thus, findings in rodent models cannot always be successfully translated to humans. Furthermore, though the zinc supplementation studies reported robust protective effects against ethanol teratogenicity, it should be noted that these studies were conducted by the same authors, making this a notable limitation. Finally, in the few existing human studies and animal models, there are discrepancies regarding the exposure and treatment dosage. Within the preclinical ethanol-exposed animal models, there was a significant variation in the amount of ethanol administered, which could be a factor that affected the variations in outcomes. For the four studies on alcohol consumption, the authors did not have the same consensus or definition of “heavy drinking”. Incongruent definitions of “heavy drinking” does not allow for the cross-study comparison on the relative effects of nutrient supplementation on offspring outcomes. The variability of nutrient dosing across the studies further limits the success of the protective effects of choline. Within the same four studies that examined concurrent multivitamin and choline supplementation, two of the studies did not confer protective effects against ethanol use in children through 12 months of age. This may be a result from an inadequate treatment dosage. More specifically, Coles et al. [59] and Kable et al. [61] supplemented 750 mg of choline paired with a multivitamin and did not find any protective effects through 12 months of age. On the other hand, the two studies [60,63] found ameliorating effects of choline through 12 months of age using the supplementation of choline alone, with a dosage of 2 g of choline per day. This indicates a possible important factor of dosing in choline efficacy.

From these limitations, we identified several future directions for this area of research. First, studies that examine the influence of nutrient supplementation in pregnancies with polysubstance use are needed. Following this is the need for studies designed to identify the optimal timing of nutrient supplementation during pregnancies with substance use. Finally, a majority of studies involve rodent models, and because rodent pregnancy is accelerated in comparison to human pregnancy, the results may not be easily translated to humans. More studies involving human participants are needed, particularly studies that are not limited in the length of assessment. More specifically, there is a need for studies to follow up years after prenatal supplementation has occurred to help elucidate the long-term benefits.

## 5. Conclusions

The effects of nutrient supplementation on substance-exposed pregnancies are promising, especially regarding choline, zinc, iron, and vitamin C. Still, there is a lack of studies, and there is no consensus amongst these studies on the dosing of substance exposure and the timing of nutrient supplementation during gestation to elucidate any clear conclusions. Furthermore, thoughtfully designed human and animal studies are needed to investigate the effects of micronutrient supplementation on polysubstance use. The continuation and augmentation of this work might lead to the development of new clinical interventions that can improve fetal outcomes in the context of prenatal substance exposure, leading to potential long-term developmental benefits.

## Figures and Tables

**Table 1:** Overview of characteristics, methodology, and results from studies evaluating the effects of nutrient supplementation on maternal substance use in outcomes related to offspring development in animals.

**a.** Effects of choline on maternal ethanol consumption and offspring developmental outcomes.				
**Reference and Study Population**	**Prenatal Substance Use (PSU)**	**Prenatal Nutrition**	**Offspring Development Outcomes**	**Results**
Balaraman et al. (2017) [33] Sprague Dawley rats (*n* = 48, male and female pups)	*Intervention group*: From PDs 4–9, received 5.25 g/kg ethanol each day in milk feeding *Control group*: From PDs 4–9, received sham intubations (i.e., milk feeding without ethanol)	*Timeframe delivered*: From PDs 4–21, 100 mg/kg choline or saline control per day *Assignment*: Randomly assigned to one of the following eight groups: (1) ethanol + saline in males, (2) ethanol + choline in males, (3) sham + saline in males, (4) sham + choline in males, (5) ethanol + saline in females, (6) ethanol + choline in females, (7) sham + saline in females, and (8) sham + choline in females	*Age at assessment*: PDs 4–9 *Outcomes assessed*: Body weight	Choline supplementation did not protect against deficits to body growth in ethanol-exposed subjects
Bearer et al. (2015) [34] C57B16/J mice (*n* = 48, male and female pups)	*Intervention group*: On PD 5, received 6.0 g/kg ethanol each day *Control group*: On PD 5, received Intralipid® sham intubations	*Timeframe delivered*: From PDs 1–20, 10 μL of 18.8 mg/mL choline or saline *Assignment*: Pseudorandomly assigned to one of the following eight groups: (1) saline from PD 1–5 + ethanol + saline from PD 6–20, (2) saline from PD 1–5 + ethanol + choline PD 6–20, (3) choline from PD 1–5 + ethanol + saline from PD 6–20, (4) choline from PD 1–5 + ethanol + choline from PD 6–20, (5) saline from PD 1–5 + sham + saline from PD 6–20, (6) saline from PD 1–5 + sham + choline from PD 6–20, (7) choline from PD 1–5 + sham + saline from PD 6–20, and (8) choline from PD 1–5 + sham + choline from PD 6–20	*Age at assessment*: PD 30 *Outcomes assessed*: Body weight Balance and coordination, as measured using the Dowel Test	Choline supplementation had no effect on body weight For both sexes, choline supplementation before and after ethanol exposure increased balance and coordination (*p* < 0.001) In males, choline supplementation *prior* to ethanol exposure increased balance and coordination (*p* = 0.009) Choline supplementation *after* ethanol exposure did not significantly increase performance on balance and coordination in males (*p* = 0.522) In females, choline supplementation *prior* to ethanol did not reach statistical significance for balance and coordination performance (*p* = 0.056) Choline supplementation *post* ethanol exposure had no effect on balance and coordination in females
Birch et al. (2016) [35] Suffolk ewes (*n* = 44, and their male and female lambs)	*Intervention group*: From GDs 4–41, received 2.5 g/kg ethanol on three consecutive days per week, followed by four days without treatment *Control group*: From GDs 4–41, received isotonic saline (0.9%) equal in volume to the ethanol infusions	*Timeframe delivered*: From GD 4 to term, 10 mg/kg/day choline *Assignment*: Randomly assigned to one of the following five groups: (1) normal untreated control, (2) saline control, (3) saline control + choline, (4) ethanol, and (5) ethanol + choline	*Age at assessment*: 6 months *Outcomes assessed*: Body weight at birth and 6 months Whole brain, cerebellar, and pituitary volumes	Choline supplementation had no protective effects on reductions in body weight and whole brain, cerebellar, and pituitary volumes in ethanol-exposed subjects
Bottom et al. (2020) [36] CD1 mice (*n* = 98? (authors did not provide the total number), male and female pups)	*Intervention group*: Throughout gestation, drank water with 25% ethanol *Control group*: Throughout gestation, drank water	*Timeframe delivered*: Throughout gestation, 642 mg/L choline *Assignment*: Randomly assigned to one of the following four groups: (1) water (control), (2) 25% ethanol in water, (3) 25% ethanol in water + choline, and (4) choline in water	*Age at assessment*: PD 0 and PD 20 *Outcomes assessed*: Body and brain weights at PD 0 Cortical length Ability to integrate sensory inputs and motor outputs, as measured using the Ledge test at PD 20 Anxiety-like behaviors, as measured using the Suok test at PD 20	Choline supplementation prevented reductions in body weight (*p* < 0.01), brain weight (*p* < 0.01), and cortical length (*p* = 0.049) Choline supplementation fully prevented deficits in motor function; choline-supplemented subjects took fewer missteps (*p* = 0.0002) and fewer falls (*p* = 0.036) compared to ethanol-exposed subjects Choline supplementation partially ameliorated anxiety-like behaviors in ethanol-exposed subjects (latency to leave center: H = 8.196, *p* = 0.042; directed exploration: H = 16.61, *p* = 0.001; rearing/grooming: H = 15.33, *p* = 0.002)
Carugati et al. (2022) [37] Suffolk ewes (*n* = 56, and their male and female lambs)	*Intervention group*: From GDs 4–41, received either 1.75 or 2.5 g/kg ethanol *Control group*: From GDs 4–41, received isotonic saline (0.9% *w*/*v*)	*Timeframe*: Throughout gestation, 10 mg/kg of oral choline *Assignment*: Randomly assigned to one of the following seven treatment groups: (1) normal control group, (2) saline control + placebo, (3) saline control + choline, (4) 1.75 g/kg ethanol + placebo, (5) 1.75 g/kg ethanol + choline, (6) 2.5 g/kg ethanol + placebo, and (7) 2.5 g/kg ethanol + choline	*Age at assessment*: At birth and 6 months *Outcomes assessed*: Birth and brain weights	Choline supplementation did not significantly affect birth or brain weights in ethanol-exposed subjects
Goeke et al. (2018) [38] Sprague Dawley rats (*n* = 31, male and female pups)	*Intervention group*: From PDs 4–9, received 5 g/kg/day ethanol *Control group*: From PDs 4–9, received sham intragastric intubations	*Timeframe delivered*: From PDs 4–9, 100 mg/kg choline or saline *Assignment*: Randomly assigned to one of the following five groups: (1) sham intubation + saline, (2) sham intubation + choline, (3) ethanol intubation + saline, (4) ethanol intubation + choline, and (5) untreated control	*Age at assessment*: PD 9 *Outcomes assessed*: Body weight	No significant differences in body weight across all treatment groups on PD 4; animals in all groups gained weight during the treatment window from PD 4 to PD 9
Hunt et al. (2014) [39] Sprague Dawley rats (*n* = 9 treatment litters and 10 control litters with 8–10 pups per litter; offspring sex was not reported)	*Intervention group*: From PDs 4–9, received 5.0 g/kg/day ethanol *Control group*: From PDs 4–9, sham controls received the tube-insertion procedure, but were not given any fluid	*Timeframe delivered*: From PDs 4–20, 18.8 mg/mL choline or saline *Assignment*: Assigned to one of the following four groups: (1) ethanol + choline, (2) ethanol + saline, (3) sham + choline, and (4) sham + saline	*Age at assessment*: PDs 4–9 and 20 for body weight, PD 23 for heart-rate-orienting response and response habituation *Outcomes assessed*: Body weight Form and magnitude of heart-rate-orienting response Habituation of orienting response	Choline supplementation did not protect against observed reductions in body weight in ethanol-exposed subjects No effect of choline supplementation on the form or magnitude of the heart-rate-orienting response or on habituation of orienting response
Kwan et al. (2021) [40] C57BL/6J mice (*n* = 32 litters, male and female fetuses)	*Intervention group*: From EDs 8.5–17.5, received 3.0 g/kg/day ethanol *Control group*: From EDs 8.5–17.5, received a single 4.20 g/kg gavage of maltodextrin	*Timeframe delivered*: From EDs 8.5–17.5, 100 mg/kg choline or saline *Assignment*: Randomly assigned to one of the following four groups: (1) sham, (2) ethanol, (3) sham + choline, and (4) ethanol + choline	*Age at assessment*: ED 17.5 *Outcomes assessed*: Body, brain, and liver weights Fetal brain-to-body weight ratio Fetal liver-to-body weight ratio Brain-to-liver-weight ratio	In males, choline supplementation did not have significant effects on body weight (*p* = 0.353), brain weight (*p* = 0.653), fetal brain-to-body weight ratio (0.497), liver weight (*p* = 0.973), fetal liver-to-body weight ratio (*p* = 0.282), or fetal brain-to-liver weight ratio (*p* = 0.536) In females, choline supplementation did not have significant effects on body weight (*p* = 0.489), brain weight (*p* = 0.673), brain-to-body weight ratio (0.066), or fetal liver-to-body weight ratio (*p* = 0.078) In females, choline supplementation mitigated reductions in liver weights such that the choline-supplemented group did not differ from the control (*p* = 0.31) or control + choline groups (*p* = 0.17) Choline supplementation mitigated high brain-to-liver weight ratio (*p* = 0.002) in females
Monk et al. (2012) [41] Sprague Dawley rats (*n* = 53, male pups only)	*Intervention group*: From PDs 4–9, received 5.25 g/k/day ethanol *Control group*: From PDs 4–9, received sham intubations	*Timeframe delivered*: From PDs 4–30, 100 mg/kg/day choline or saline *Assignment*: Randomly assigned to one of the following four groups: (1) ethanol + choline, (2) ethanol + saline, (3) control + choline, and (4) control + saline	*Age at assessment*: PDs 4–30 and PDs 30–33 *Outcomes assessed*: Body weight on PDs 4–30 Hyperactivity, as measured using the open field test on PDs 30–33	Choline supplementation had no effect on body weight Choline supplementation reduced hyperactivity levels in ethanol-exposed subjects (*p* < 0.05)
Otero et al. (2012) [42] Long-Evans rats (*n* = 120, male and female pups)	*Intervention group*: From PDs 2–10, received 3.0 g/kg/day ethanol *Control groups*: From PDs 2–10, intubated without alcohol and a nontreated control group	*Timeframe delivered*: From PDs 2–20, 100 mg/kg choline or saline *Assignment*: Quasi-randomly assigned to one of the following five groups: (1) ethanol + choline, (2) ethanol + saline, (3) intubated control + choline, (4) intubated control + saline, and (5) nontreated control	*Age at assessment*: PD 2–21 *Outcomes assessed*: Body weight	Choline supplementation did not protect against deficits in growth in ethanol-exposed subjects
Sawant et al. (2019) [43] Suffolk ewes (*n* = 49, offspring sex was not reported)	*Intervention group*: From GDs 4–41, received either 1.75 g/kg/day or 2.25 g/kg/day ethanol *Control group*: From GDs 4–41, received 0.9% isotonic saline infusions intravenously	*Timeframe delivered*: From GD 4 until term, 10 mg/kg per day *Assignment*: Randomly assigned to one of the following six groups: (1) saline + placebo control, (2) saline + choline, (3) 1.75 g/kg/day ethanol + placebo, (4) 1.75 g/kg/day ethanol + choline, (5) 2.25 g/kg/day ethanol, and (6) 2.25 g/kg/day ethanol + choline	*Age at assessment*: GD 76 *Outcomes assessed*: Fetal frontothalamic distance, mean orbital diameter, interorbital distance, mean lens diameter, thalamic width, and femoral and humerus lengths	Choline supplementation protected against decreases in brain fetal frontothalamic distance (*p* = 0.013) Choline supplementation had no significant effect on mean orbital diameter (*p* > 0.05) or interorbital distance in ethanol-exposed subjects (*p* = 0.101) Choline supplementation significantly increased fetal mean lens diameter in ethanol-exposed subjects (*p* < 0.001) Choline supplementation significantly decreased fetal thalamic width (*p* = 0.043) There was no significant interaction between choline and ethanol use on femoral and humerus length; choline supplementation increased femoral (*p* = 0.002) and humerus (*p* = 0.011) and lengths across all groups
Steane et al. (2021) [44] Sprague Dawley rates (*n* = 57, male and female fetuses)	*Intervention group*: From 4 days prior to conception and 4 days after conception, received a liquid diet containing 12.5% EtOH (*v*/*v*) *Control group*: From 4 days prior to conception and 4 days after conception, received a control liquid diet	*Timeframe delivered*: From GDs 5–20, 1.6 g choline/kg or 2.6 g choline/kg with one group increased to 7.2 g choline/kg from GDs 10–20 *Assignment*: Randomly assigned to one of the following six groups: (1) liquid control + choline (1.6 g/kg), (2) ethanol + choline (1.6 g/kg), (3) liquid control + choline (2.6 g/kg), (4) ethanol + choline (2.6 g/kg), (5) liquid control + choline (2.6 g/kg from GDs 5–10, followed by 7.2 g/kg chow from GDs 10–20), and (6) ethanol + choline (2.6 g/kg chow from GDs 5–10, followed by 7.2 g/kg chow from GDs 10–20)	*Age at assessment*: GD 20 *Outcomes assessed*: Body, liver, and heart weights	Though not statically significant, the reduction in body weight with the 1.6 g/kg choline diet was ~8% in males and ~7% in females, compared to 2–4% with the 2.6 g/kg choline and 7.2 g/kg choline groups in ethanol-exposed males (*p* = 0.30) and females (*p* = 0.77) Choline supplementation did not have significant effects on liver weights in males (*p* = 0.77) or females (*p* = 0.85) Choline supplementation did not have significant effects on heart weights in males (*p* = 0.88), but there was a significant effect on heart weights in females (*p* = 0.01), but only for the 1.6 g/kg choline group
Thomas et al. (2000) [45] Sprague Dawley rats (*n* = 78, male and female pups from 13 dams)	*Intervention group*: From GDs 6–20, received a liquid diet containing 35% ethanol-derived calories *Control groups*: From GDs 6–20, received a liquid isocaloric maltose-dextrin and a nontreated control group was fed regular lab chow ad lib	*Timeframe delivered*: From PDs 2–7, 25 mg choline chloride/mL saline *Assignment*: Randomly assigned to one of the following nine groups: (1) ethanol + choline, (2) ethanol + saline, (3) ethanol + lab chow, (4) liquid control + choline, (5) liquid control + saline, (6) liquid control + lab chow, (7) nontreated control + choline, (8) nontreated control + saline, and (9) nontreated control + lab chow	*Age at assessment*: PD 4–21 and PD 45 *Outcomes assessed*: Body growth on PDs 4–21 Visuospatial discrimination, as measured using the T-maze task on PD 45	Choline supplementation had no effect on body growth in ethanol-exposed subjects Choline supplementation improved visuospatial discrimination acquisition in ethanol-exposed subjects (*p* < 0.01) Choline supplementation produced a relatively large improvement in delayed discrimination training performance among the ethanol-treated subjects and only mild improvement in the pair-fed and liquid controls (*p* < 0.002)
Thomas et al. (2004) [46] Sprague Dawley rats (*n* = 82, male pups only)	*Intervention group*: From PDs 4–9, received 6.6 g/kg/day ethanol *Control group*: From PDs 4–9, received isocaloric maltose-dextrin	*Timeframe delivered*: From PDs 4–30, 18.8 mg choline chloride/mL or saline *Assignment*: Randomly assigned to one of the following six groups: (1) ethanol + choline, (2) ethanol + saline, (3) sham intubation + choline, (4) shame intubation + saline, (5) normal lactation control + choline, and (6) normal lactation control + saline	*Age at assessment*: PD 35–37 *Outcomes assessed*: Motor coordination, as measured via maximum gap successfully traversed, ratio of successful traversals to total traversals, and number of trials to first successful traversal via the Parallel Bars task	Choline supplementation did not attenuate impairments to motor coordination, measured via maximum gap successfully traversed, ratio of successful traversals to total traversals, or number of trials to first successful traversal, in ethanol-exposed subjects
Thomas et al. (2004) [47] Sprague Dawley rats (*n* = 85, male pups only)	*Intervention group*: From PDs 4–9, received 6.6 g/kg/day ethanol *Control group*: From PDs 4–9, received isocaloric maltose-dextrin	*Timeframe delivered*: From PDs 4–30, 18.8 mg choline chloride/mL or saline *Assignment*: Randomly assigned to one of the following six groups: (1) ethanol + choline, (2) ethanol + saline, (3) intubated control + choline, (4) intubated control + saline, (5) normal lactation control + choline, and (6) normal lactation control + saline	*Age at assessment*: PD 31–34 and 40–42 *Outcomes assessed*: Body weight Activity level on PDs 31–34 Spatial discrimination serial reversal learning as measured by number of trials to the first successful criterion, total number of successful discriminations achieved, and number of errors via the T-maze task, on PDs 40–42	Choline supplementation did not have any significant effects on body weight in ethanol-exposed subjects Choline supplementation significantly reduced activity levels in ethanol-exposed subjects compared to controls (*p* < 0.01) There were no statistically significant effects of ethanol or choline on the number of trials to the first successful criterion or the total number of successful discriminations achieved Choline supplementation significantly reduced the number of errors committed on the spatial discrimination learning task among ethanol-treated subjects (*p* < 0.05)
Thomas et al. (2009) [48] Sprague Dawley rats (*n* = 72, male and female pups)	*Intervention group*: From GDs 5–20, received 6.0 g/kg/day in a 28.5% (*v*/*v*) ethanol solution (0.02675 mL/g body weight) *Control group*: From GDs 5–20, pair-fed dams received isocaloric maltose-dextrin, and ad lib control dams received a vehicle full of saline	*Timeframe delivered*: From GDs 5–20, 250 mg choline/kg/day or saline *Assignment*: Randomly assigned to one of the following six groups: (1) ethanol + choline, (2) ethanol + saline, (3) pair-fed intubation + choline, (4) pair-fed intubation + saline, (5) ad lib control + choline, and (6) ad lib control + saline	*Age at assessment*: PD 1–21 and PD 2–20 *Outcomes assessed*: Body (PDs 1–21) and brain weights Eye opening and incisor emergence Series of reflex development tasks to examine sensorimotor maturation including righting reflex, geotactic reflex, cliff avoidance, grip strength, and hindlimb coordination	Choline supplementation significantly attenuated alcohol-related birth weight reductions (*p* < 0.05) and brain weights (*p* < 0.05) Choline supplementation advanced incisor emergence across both ethanol and control groups (*p* < 0.05) There was no significant interaction between ethanol and choline for eye opening and grip strength Choline supplementation attenuated effects of righting reflex responses such that they were not significantly different from that observed in control groups (Fisher’s *p*’s < 0.05) Choline supplementation significantly mitigated deficits in the negative geotactic reflex observed in ethanol-exposed subjects (Fisher *p*’s < 0.05) Choline-supplemented subjects exposed to ethanol performed significantly similarly to control subjects on the behavioral measure of cliff avoidance (*p* < 0.01) Choline supplementation showed a tendency to reduce the deficit in decreased hindlimb coordination in ethanol-exposed subjects; no significant interaction was observed
Thomas et al. (2010) [49] Sprague Dawley rats (*n* = 71 male and female litters)	*Intervention group*: From GDs 5–20, received 6.0 g/kg/day (28.5% *v*/*v*) ethanol *Control group*: From GDs 5–20, received an isocaloric maltose-dextrin solution	*Timeframe delivered*: From GDs 5–20, 250 mg choline/kg/day or saline *Assignment*: Randomly assigned to one of the following six groups: (1) ethanol + choline, (2) ethanol + saline, (3) pair-fed isocaloric maltose-dextrin solution + choline, (4) pair-fed isocaloric maltose-dextrin solution + saline, (5) ad libitum control + choline, and (6) ad libitum control + saline	*Age at assessment*: PD 15–17, PD 28–32, PD 30–32, PD 39–41, PD 45–52 and PD 65–66 *Outcomes assessed*: Body weight Exploratory behavior, a measure of natural exploratory and foraging behavior that depends on hippocampal cholinergic functioning, as measured using T-maze spontaneous alternation behavioral task Motor coordination, as measured using success ratio and maximum width traversed via the parallel bars task Spatial learning and working memory, as measured using the Morris water maze task	Across all treatment groups, choline supplementation significantly increased body weight at PD 28 (*p* < 0.0001) and 45 (*p* < 0.05), but not at PD 30 (*p* = 0.13) Choline-supplemented subjects alternated at significantly higher rates; ~75% of subjects compared to ~35% of ethanol-exposed subjects not supplemented with choline during the spontaneous alteration task Choline supplementation did not affect the motor performance on the parallel bar task, including success ratio and maximum width traversed (all *p*-values >0.1) Choline supplementation significantly mitigated impairments to spatial working memory (*p* < 0.05) The interaction of choline with prenatal ethanol exposure did not reach statistical significance for the spatial learning task
Wagner and Hunt (2006) [50] Sprague Dawley rats (*n* = 85, male and female pups)	*Intervention group*: From PDs 4–9, received 5.25 g/kg/day ethanol *Control group*: From PDs 4–9, received sham intubations	*Timeframe delivered*: From PDs 4–20, 0.10 mL of an 18.8 mg/mL solution of choline, chloride, or saline *Assignment*: Randomly assigned to one of the following eight groups: (1) ethanol + choline + delay conditioning, (2) ethanol + choline + trace conditioning, (3) ethanol + saline + delay conditioning, (4) ethanol + choline + trace conditioning, (5) sham + choline + delay conditioning, (6) sham + choline + trace conditioning, (7) sham + saline + delay conditioning, and (8) sham + choline + trace conditioning	*Age at assessment*: PD 4–9, 15, and 20 and PD 30 *Outcomes assessed*: Body weight on PDs 4–9, 15, and 20 Conditioned stimulus-elicited freezing on PD 30	Choline supplementation had no effect on body weights Choline supplementation completely reversed the deficit in conditioned stimulus freezing for the trace conditioning groups (*p* < 0.01) None of the groups given delayed conditioning trials differed in conditioned stimulus freezing
**b.** Effects of zinc on maternal ethanol consumption and offspring developmental outcomes.				
**Reference and Study Population**	**Prenatal Substance Use (PSU)**	**Prenatal Nutrition**	**Offspring Development Outcomes**	**Results**
Summers et al. (2006) [51] C57BL/6J mice (*n* = 72, male and female pups)	*Intervention group*: On GD 8, received 25% ethanol in 0.85% saline *v*/*v* (0.015 mL/g) intraperitoneally twice *Control group*: On GD 8, received saline injections	*Timeframe delivered*: On GD 8, 0.25 mL zinc *Assignment*: Assigned to one of the following three groups: (1) saline, (2) ethanol, and (3) ethanol + zinc	*Age at assessment*: PD 7 and 21, 56–60, and 70–71 *Outcomes assessed*: Body weight on PD 7, 21, and 55 Spatial learning and memory, as measured via escape latency, number of correct trials and errors via the cross-maze water escape task	Zinc supplementation increased body weights on PD 55 in males but not females (*p* = 0.001) Choline supplementation attenuated effects of ethanol on spatial memory on all parameters, including shorter escape latencies, more correct trials, and fewer incorrect entries (*p* < 0.05)
Summers et al. (2008) [52] C57BL/6J mice (*n* = 24/treatment, male and female pups)	*Intervention group*: On GD 8, received 25% ethanol (0.015 mL/g) injections twice *Control group*: On GD 8, received saline injections	*Timeframe delivered*: From GDs 1–18, 200 µg/g zinc-supplemented diet, or 35 µg/g zinc for the control group *Assignment*: Assigned to one of the following four groups: (1) saline + control diet (35 µg/g zinc), (2) ethanol + control diet (35 µg/g zinc), (3) saline + zinc -supplemented diet (200 µg/g zinc), and (4) ethanol + zinc-supplemented diet (200 µg/g zinc)	*Age at assessments*: PD 3, 21, 40, 60–66, 78, 105, 120, and 121 *Outcomes assessed*: Body weight and length on PD 3, 21, and 40 Object recognition memory, as measured by the object recognition memory tasks Spatial learning and memory impairments, as measured via escape latency, number of correct trials, and number of errors via the cross-maze water escape task	Zinc supplementation had no effect on body weight or length Zinc-supplemented subjects performed at the level of control offspring for the cross-maze water escape and object recognition memory tasks, while the ethanol only group performed worse than all other groups (*p* < 0.0001) Zinc-supplemented subjects performed to the level of saline-treated mice with shorter escape latencies for spatial memory and increased correct trials compared with mice treated with ethanol alone in the cross-maze water escape task (*p* < 0.05)
Summers et al. (2009) [53] C57BL⁄6J mice (*n* = 309, males and females)	*Intervention group*: On GD 8, received 25% (0.015 mL/g) ethanol injections *Control group*: On GD 8, received saline injections	*Timeframe delivered*: From GDs 1–18, 200 mg zinc⁄kg or 35 mg zinc/kg for the control group *Assignment*: Assigned to one of the following four groups: (1) saline + control diet (35 mg zinc⁄kg), (2) ethanol + control diet (35 mg zinc⁄kg), (3) saline + zinc-supplemented diet (200 mg zinc⁄kg), and (4) ethanol + zinc-supplemented diet (200 mg zinc⁄kg)	*Age at assessment*: GD 18 to PD 60 *Outcomes assessed*: Postnatal growth and survival Fetal dysmorphology	Cumulative postnatal mortality was significantly higher in offspring exposed to ethanol alone (35% deaths) compared to all other treatment groups (13.5 to 20.5% deaths) Zinc supplementation decreased the number of deaths from birth to PD 3 from 25 in the ethanol only group to 11 across all three other groups Zinc supplementation reduced the occurrence of stillbirths from 7 in the ethanol only group to 1 in the ethanol + zinc group, but did not reach significance Zinc supplementation reduced the incidences of physical abnormalities from 26% in the ethanol only group to 12% in the ethanol + zinc group (*p* < 0.05)
**c.** Effects of vitamin E on maternal ethanol consumption and offspring developmental outcomes.				
**Reference and Study Population**	**Prenatal Substance Use (PSU)**	**Prenatal Nutrition**	**Offspring Development Outcomes**	**Results**
Marino et al. (2004) [54] Long–Evans rats (*n* = 146, male and female pups) This study consisted of two cohorts. Cohort one outcomes were body weight and the Morris water maze. Cohort two outcomes were related to Western blot staining. We only reviewed cohort 1.	*Intervention group*: From PDs 7–9, received 5.25 g/kg/day ethanol in 27.8 mg/kg volume of milk *Control groups*: From PDs 7–9, the control was intubated without any liquids, while the nontreated control were not intubated	*Timeframe Delivered*: On PDs 6–9, 2.0 g/kg vitamin E in 13.9 mL/kg volume of milk *Assignment*: Assigned to one of the following five groups: (1) only ethanol, (2) ethanol + vitamin E, (3) intubated control, (4) intubated + vitamin E, and (5) nontreated control	*Age at assessment*: PD 6–30 *Outcomes assessed*: Body weight Spatial navigation, as measured by escape latency, duration in probe quadrant, number of probe crossings, and escape latency to reach visible platform via the Morris water maze task	Vitamin E supplementation did not mitigate reductions in body weight (*p* < 0.001) Vitamin E treatment did not attenuate significantly slower spatial navigation latencies in the ethanol-exposed animals (*p* < 0.001) No significant effect of vitamin E on the number of probes crossing in ethanol-exposed subject (*p* = 0.650), duration in probe quadrant (*p* = 0.157), or escape latency to reach visible platform (*p* = 0.868)
Tran et al. (2005) [55] Long–Evans rats (*n* = 44, male and female pups)	*Intervention group*: From PDs 4–9, received 2.625 g/kg/day ethanol, 4 intubations per day *Control group*: From PDs 4–9, received sham intubations	*Timeframe delivered*: From PDs 4–9, 12.26 mg vitamin E/kg/feeding for 4 daily feedings *Assignment*: Assigned to one of the following five groups: (1) ethanol + milk, (2) ethanol + vitamin E, (3) only vitamin E, (4) sham intubations, and (5) nontreated control	*Age at assessment*: PD 26–33 *Outcomes assessed*: Body weight Eyeblink classic conditioning	There were no interactive effects for body weight Vitamin E did not improve eyeblink conditioning performance (*p* < 0.0001) Vitamin E supplementation did not protect against reductions in eyeblink performance; mean percent conditioned responses and amplitude of conditioned responses were significantly lower than in the control groups (*p* < 0.05)
**d.** Effects of fatty acids on maternal ethanol consumption and offspring developmental outcomes.				
**Reference and** **Study Population**	**Prenatal Substance Use (PSU)**	**Prenatal Nutrition**	**Offspring Development Outcomes**	**Results**
Abel and Reddy (1997) [56] Sprague Dawley rats (*n* = 120–180 (authors did not provide the total number), male and female pups)	*Intervention group*: From GD 8–20, intubated with 5.0 or 3.0 g/kg ethanol *Control group*: From GD 8–20, intubated with a saline vehicle	*Timeframe delivered*: From GDs 8–20, diet high in saturated or polyunsaturated fat with no vitamin E or zinc (authors did not provide exact amount) *Assignment*: Randomly assigned to one of the following twelve groups: (1) saturated fat diet + 5.0 g/kg ethanol, (2) saturated fat diet + 3.0 g/kg ethanol, (3) saturated fat diet + saline intubation, (4) saturated fat diet + non-intubated control, (5) unsaturated fat diet (with a low dietary content of vitamin E and zinc) + 5.0 g/kg ethanol, (6) unsaturated fat diet (with a low dietary content of vitamin B and zinc) + 3.0 g/kg ethanol, (7) unsaturated fat diet (with a low dietary content of vitamin E and zinc) + saline intubation, (8) unsaturated fat diet + non-intubated control, (9) standard rodent diet + 5.0 g/kg ethanol, (10) standard rodent diet + 3.0 g/kg ethanol, (11) standard rodent diet + saline intubation, and (12) standard rodent diet + non-intubated control	*Age at assessment*: Birth and PD 20 *Outcomes assessed*: Birth weight Locomotor activity Head-dipping behavior	There were no significant interactive effects of saturated/unsaturated fat supplementation on birth weight Saturated-fat-supplemented subjects were less active than the pair-fed controls (*p* < 0.001) Saturated fat supplementation produced a significant decrease in head-dipping behavior, indicating a high anxiety-like state (*p* < 0.01) The polyunsaturated fat and low vitamin E and zinc diet did not modify ethanol’s effects significantly for locomotor activity or head-dipping behavior
Wainwright et al. (1985) [57] B6D2F1 mice (*n* = 32–48, male and female pups)	*Intervention group*: From GDs 7–17, received liquid diets containing 25% ethanol-derived calories (4.7 mL) *Control group*: From GDs 7–17, received liquid diet containing 6.25 g of isocaloric sucrose	*Timeframe delivered*: From GDs 7–17, either 20 mg/kg, 120 mg/kg, or 200 mg/kg gamma-linolenic acid *Assignment*: Randomly assigned to one of the following eight groups: (1) ethanol + gamma-linolenic acid (20 mg/kg), (2) ethanol + gamma-linolenic acid (120 mg/kg), (3) ethanol + gamma-linolenic acid (200 mg/kg), (4) ethanol + arachidonic acid control (200 mg/kg), (5) ethanol + coconut oil control, (6) ethanol + safflower oil control, (7) sucrose intubations + safflower oil, and (8) lab chow + no fatty acid supplementation	*Age at assessment*: PD 22, 32, and 50 *Outcomes assessed*: Body and brain weights Behavioral development on PD 32, including righting reflex, cliff aversion, forelimb and hindlimb grasp reflex, Vibrissa placing reflex, level screen test, vertical screen test, screen climbing test, pole grasp, forelimb and hindlimb stick grasp, opening of both eyes, visual placing reflex, auditory startle response Behavior was measured in an open field on PD 50 including the following behavioral categories: animal in quadrupedal movement, animal sniffing the air, animal sniffing an object, animal rearing, animal grooming, animal freezing	Gamma-linolenic acid supplementation did not prevent deficits in body and brain weights There were no interactive effects of gamma-linolenic acid supplementation on any behavioral development outcomes of interest
**e.** Effects of iron on maternal ethanol consumption and offspring developmental outcomes.				
**Reference and Study Population**	**Prenatal Substance Use (PSU)**	**Prenatal Nutrition**	**Offspring Development Outcomes**	**Results**
Helfrich et al. (2022) [58] Long–Evans rats (*n* = 140 litters, (authors did not provide the total number), male and female pups)	*Intervention group*: From GDs 13.5–19.5, received 5.0 g/kg/day of ethanol *Control group*: From GDs 13.5–19.5, received a 43.8% maltodextrin solution in water	*Timeframe delivered*: From GDs 12.5–19.5, received 6 mg/kg elemental iron *Assignment*: Randomly assigned to one of the following four groups: (1) non-intubated control + water, (2) ethanol + water, (3) non-intubated control + iron, and (4) ethanol + iron	*Age at assessment*: Birth and PD 20 *Outcomes assessed*: Birth weight Liver weight Brain weight Heart weight	Iron supplementation significantly improved absolute brain weights in male pups alone (*p* = 0.014) There were no interactive effects of iron supplementation on any other physical developmental outcomes of interest

GD = gestational day, PD = postnatal day, ED = embryonic day.

**Table 2:** Overview of characteristics, methodology, and results from studies evaluating the main interaction effects of substance use and nutrient supplementation on outcomes related to offspring development in humans.

**a.** Effects of choline on maternal alcohol consumption and offspring developmental outcomes.				
**Reference and Study Population**	**Prenatal Substance Use**	**Prenatal Nutrition**	**Offspring Development Outcomes**	**Results**
Coles et al. (2015) [59] Infants (*n* = 367, males and females)	*Intervention group*: “Heavy” drinkers (*n* = 301), defined as having at least weekly binge drinking episodes (5+ drinks), at least five episodes in which they consumed 3–4 standard drinks, or at least ten episodes in which they consumed 1–2 standard drinks either in the month around conception or the most recent month of pregnancy *Control group*: Nondrinking women (*n* = 313), meeting screening criteria (i.e., no binge episodes, minimal or no alcohol consumption in the month around conception, and no drinking in the most recent month of pregnancy)	*Timeframe delivered*: From the first prenatal visit (average 19 weeks) until delivery, 750 mg choline *Assignment*: Alcohol-using and nondrinking women were randomized to one of the following three groups: (1) no multivitamin supplement provided, but recommended (*n* = 176), (2) multivitamin supplement provided (*n* = 96), and (3) multivitamin supplement + choline provided (*n* = 95)	*Age at assessment*: Birth and 6 months of age *Outcomes assessed*: Birth weight and length and head circumference Bayley Scales of Infant Development 2nd Edition (BSID-II) measures current mental development (problem solving/prelinguistic development) and psychomotor development (fine/gross motor skills) yielding standardized scores (Mental Development Index: MDI; Psychomotor Development Index: PDI) Behavioral rating in the BSID-II of orientation/engagement, emotional regulation, motor quality, and total behavior quality	Supplementation (multivitamin or multivitamin with choline) did not have any effect on birth weight, length, or head circumference Choline supplementation approached significance (*p* = 0.10), with those taking choline having lower scores on the Psychomotor Development Index, but did not contribute significantly to differences in the Psychomotor Development Index There was no effect of the interaction of choline with alcohol exposure on the Mental Development Index (*p* = N.S.) The multivitamin group had significantly higher scores on the Mental Development Index, which was not observed in the multivitamin + choline group (*p* < 0.03) There were no interactive effects of choline supplementation on behavioral ratings on the BSID-II including orientation/engagement, emotional reactivity, motor quality, and total behavior
Jacobson et al. (2018) [60] Infants (*n* = 62, males and females)	*Intervention group*: Heavy drinkers (*n* = 35) were recruited, defined by having an average of at least 2 standard drinks (1.0 oz absolute alcohol) per day or at least one incident of binge drinking (4 or more standard drinks/occasion) *Control group*: Heavy drinkers (*n* = 35); did not receive choline supplement	*Timeframe delivered*: Time of enrollment (23rd week of gestation) until delivery, 2 g/day choline *Assignment*: Heavy drinkers were randomly assigned to one of the following two groups: (1) choline supplement and (2) placebo pill	*Age at assessment*: 6.5 months and 12 months *Outcomes assessed*: Somatic growth Recognition memory and processing speed, as measured using the Fagan Test of Infant Intelligence Eyeblink conditioning Fetal alcohol spectrum disorder (FASD) or partial fetal alcohol syndrome (PFAS) diagnosis	Choline supplementation showed significantly greater increases in weight (*p* = 0.009) and head circumference (*p* = 0.006) Choline supplementation had a significantly greater increase in percent condition eyeblink responses than the placebo group (*p* < 0.01) between 6.5 and 12 months of age There was a non-significant increase in the proportion of infants meeting the EBC in the choline group (*p* = 0.090), but when those whose mothers with poor adherence (< 20%) were excluded, the increase in the proportion meeting EBC was significant (*p* = 0.036) Choline-supplemented infants performed more optimally on the Fagan Test of Infant Intelligence at 12 months, with higher novelty preference scores, indicating better visual recognition memory function (d = 0.62, *p* < 0.05) Choline supplementation did not improve the proportion of infants diagnosed with FASD/PFAS. In the choline group, 8 infants were diagnosed with FASD and 2 were diagnosed with PFAS (32.3%), while in the placebo group, only 5 infants were diagnosed with FASD and 2 were diagnosed with PFAS (22.6%) (*p* = 0.393) For consideration, this study reports a high level of cigarette use (1/4 pack/day), and 4 participants reported use of methamphetamine later in pregnancy
Kable et al. (2015) [61] Infants (*n* = 168, males and females)	*Intervention group*: Women (*n* = 119) who reported at least weekly binge drinking episodes (5+ drinks), at least five episodes in which they consumed 3–4 standard drinks, or at least 10 episodes in which they consumed 1–2 standard drinks either in the month around conception or in the most recent month of pregnancy *Control group*: Women (*n* = 136) who reported no binge drinking episodes, minimal or no alcohol consumption in the month around conception, and no continued drinking during pregnancy	*Timeframe delivered*: From first prenatal visit until delivery, 750 mg choline *Assignment*: Alcohol-using and nondrinking women were randomized to one of the following three groups: (1) multivitamin supplement recommended but not provided (*n* = 81), (2) multivitamin supplement provided (*n* = 50), and (3) multivitamin supplement + choline provided (*n* = 37)	*Age at assessment*: Birth and 6–12 months *Outcomes assessed*: Birth weight and length Head circumference Cardiac-orienting responses during a habituation/dishabituation learning paradigm to assess neurophysiological encoding and memory of environmental events	Choline supplementation did not protect against reductions in birth weight, length, and head circumference Choline supplementation plus multivitamin did not significantly affect cardiac-orienting responses to the auditory stimuli in alcohol-exposed pregnancies There were no interactive effects of choline on latency response in the visual habituation tasks Choline supplementation resulted in a greater change in heart rate on the visual habituation task across ethanol-exposed and control groups (*p* < 0.001) Change in choline level from the baseline to third trimester timepoint was positively related to HR during the habitation task (*p* < 0.05), but not the dishabituation task or latency of the response during either task for both ethanol-exposed and control groups This study reports that those consuming alcohol had significantly higher cigarette use (*p* < 0.005)
Kable et al. (2022) [62] Infants (*n* = 243, males and females)	*Intervention group*: Women (*n* = 141) who reported at least weekly binge drinking episodes (5+ drinks), at least five episodes in which they consumed 3–4 standard drinks, or at least 10 episodes in which they consumed 1–2 standard drinks either in the month around conception or in the most recent month of pregnancy *Control group*: Women (*n* = 225) who reported no binge drinking episodes, minimal or no alcohol consumption in the month around conception, and no continued drinking during pregnancy	*Timeframe delivered*: From first prenatal visit until delivery, 750 mg choline *Assignment*: Alcohol-using and nondrinking women were randomized to one of the following three groups: (1) multivitamin supplement recommended but not provided (*n* = 114), (2) multivitamin supplement provided (*n* = 52), and (3) multivitamin supplement + choline provided (*n* = 77)	*Age at assessment*: Mean age 3.96 years *Outcomes assessed*: Reaction time involving the child making a response to a series of chromatic pictures presented on a computer screen	Prenatal choline supplementation did not improve reaction time in preschool-aged children exposed to alcohol in utero
Warton et al. (2021) [63] Infants (*n* = 50, males and females)	*Intervention group*: Heavy drinkers (*n* = 27), defined as having an average of at least 2 standard drinks (1.0 oz absolute alcohol) per day or at least one incident of binge drinking (4 or more standard drinks/occasion) *Control group*: Heavy drinkers (*n* = 23); did not receive choline supplement	*Timeframe delivered*: Time of enrollment (23rd week of gestation) until delivery, 2 g/day choline *Assignment*: Heavy drinkers were randomly assigned to one of the following two groups: (1) choline supplement and (2) placebo pill	*Age at assessment*: 1–7 weeks of age (median age 2.8 weeks) and 12 months of age *Outcomes assessed*: Brain volumes Recognition memory and processing speed, as measured using the Fagan Test of Infant Intelligence (FTII)	Choline supplementation increased brain volume in 6 of 12 regions, namely, the left (*p* = 0.01) and right thalamus (*p* = 0.05) and left (*p* = 0.006) and right caudate (*p* = 0.004), right putamen (*p* = 0.03), and corpus callosum (*p* = 0.01) The effect of choline on FTII was significant after controlling maternal supplement adherence and birth weight (*p* = 0.03) In regions where choline effects were observed, the presence of a larger right putamen (*p* = 0.003) and corpus callosum (*p* ≤ 0.04) were associated with higher scores for Fagan Test of Infant Intelligence More mothers in the choline group reported smoking (96% vs. 70%), and cannabis use was more common in the choline group (41% vs. 9%)
**b.** Effects of vitamin C on maternal nicotine consumption and offspring developmental outcomes.				
**Reference and Study Population**	**Prenatal Substance Use**	**Prenatal Nutrition**	**Offspring Development Outcomes**	**Results**
McEvoy et al. (2014) [64] Infants (*n* = 235, males and females) Stand-alone cohort	*Intervention group*: Current smokers (≥ 1 cigarette/day) *Control group*: Pregnant nonsmokers who were not given vitamin C or a placebo (*n* = 76)	*Timeframe delivered*: Randomized at ≤22 weeks gestational age to receive 500 mg/day vitamin C *Assignment*: Pregnant current smokers were randomly assigned to one of the following two groups: (1) vitamin C supplement (*n* = 89) and (2) placebo pill (*n* = 90)	*Age at assessment*: 3 days and 12 months *Outcomes assessed*: Newborn pulmonary function at 3 days Incidence of wheezing through 12 months and pulmonary function at 12 months	Vitamin C supplementation significantly improved pulmonary function as measured by time to peak tidal expiratory flow to expiratory time, (*p* =0.006), passive respiratory system compliance (*p* = 0.012), and total passive respiratory system compliance (*p* = 0.0035) at 3 days of age Vitamin C supplementation significantly decreased the occurrence of wheezing through 1 year from 40% in the placebo group to 21% in the vitamin C group (adjusted relative risk 0.56; 95% CI 0.33, 0.95; *p* = 0.03)
McEvoy et al. (2019) [65] Infants (*n* = 225, males and females) * VCSIP cohort	*Intervention group*: Current smokers (≥ 1 cigarette/day) who were given vitamin C *Control group*: Pregnant smokers who were given a placebo	*Timeframe delivered*: Randomized at ≤22 weeks gestational age to receive 500 mg/day vitamin C *Assignment*: Pregnant current smokers were randomly assigned to one of the following two groups: (1) vitamin C supplement (*n* = 125) and (2) placebo pill (*n* = 126)	*Age at assessment*: Birth and 3 months *Outcomes assessed*: Birth weight Delivery mode Gestational age Incidence of prematurity Forced expiratory flows at 25%, 50%, and 75% of expired volumes	Vitamin C supplementation increased (but not significantly) forced expiratory flow at 75% at 3 months of age (200.7 vs. 188.7 mL/s; adjusted 95% confidence interval (CI) for difference, −3.33 to 35.64; *p* = 0.10), and a significantly increased forced expiratory flow at 50% (436.7 vs. 408.5 mL/s; adjusted 95% CI, 6.10–61.30; *p* = 0.02) when compared with those born to smokers who were randomized to receive the placebo There were no significant (*p* > 0.05) effects of vitamin C supplementation on birth weight, delivery mode, gestational age, and incidence of prematurity
McEvoy et al. (2020) [66] Infants (*n* = 213, males and females) * VCSIP cohort	*Intervention group*: Current smokers (≥ 1 cigarette/day) *Control group*: Pregnant smokers who were given a placebo	*Timeframe delivered*: Randomized at ≤22 weeks gestational age to receive 500 mg/day vitamin C *Assignment*: Pregnant current smokers were randomly assigned to one of the following two groups: (1) vitamin C supplement (*n* = 125) and (2) placebo pill (*n* = 126)	*Age at assessment*: 3 and 12 months *Outcomes assessed*: Sustained changes in forced expiratory flows at 75%, 50%, and 25–75% volume, (FEF75, FEF50, FEF25–75) and forced expired volume (FEV0.5) Incidence of wheezing	Forced expiratory flows in the vitamin C-treated group increased by 16.1% for FEF75, 11.6% for FEF50, 12% for FEF25–75, and 6.8% for FEV0.5 when compared with the placebo group at 12 months of age; however, none of them reached significance (*p* > 0.05) Vitamin C produced a persistently significant increase in the offspring’s airway function at 12 months of age in sustained forced expiratory flows at 75%, 50%, and 25% expired volume; FEF75 of 40.2 mL/sec (adjusted 95% CI 6.6, 73.8; *p* = 0.0248); FEF50 of 58.3 mL/sec (10.9, 105.8; *p* = 0.0081)); FEF25–75 of 55.1 mL/sec (9.7, 100.5; 0.0130); and FEV0.5 of 16.3 mL (1.0, 31.6; *p* = 0.174) Vitamin C supplementation did not produce any significant effects on the incidence of wheezing at 12 months of age (*p* = 0.13)
McEvoy et al. (2023) [67] Infants (*n* = 213, males and females) * VCSIP cohort	*Intervention group*: Current smokers (≥ 1 cigarette/day) *Control group*: Pregnant smokers who were given a placebo	*Timeframe delivered*: Randomized at ≤22 weeks gestational age to receive 500 mg/day vitamin C *Assignment*: Pregnant current smokers were randomly assigned to one of the following two groups: (1) vitamin C supplement (*n* = 125) and (2) placebo pill (*n* = 126)	*Age at assessment*: 5 years *Outcomes assessed*: Forced expiratory flows at 25%, 50%, and 75% of expired volumes Forced expiratory volume in 1 s Incidence of wheezing	Vitamin C supplementation significantly increased the mean measurements of forced expiratory flow by 17.2% at 25–75% of expired volumes (1.45 (0.04) vs. 1.24 (0.04) L/s; adjusted mean difference, 0.21 (95% CI, 0.13- 0.30); *p* < 0.001) Vitamin C supplementation significantly increased mean measurements of forced expiratory flow by 14.1% at 50% of expired volumes (1.59 (0.04) vs. 1.39 (0.04) L/s; adjusted mean difference, 0.20 (95% CI, 0.11 to 0.30); *p* < 0.001) Vitamin C supplementation significantly increased mean measurements of forced expiratory flow by 25.9% at 75% of expired volumes (0.79 (0.02) vs. 0.63 (0.02) L/s; adjusted mean difference, 0.16 (95% CI, 0.11 to 0.22); *p* < 0.001) Supplemental vitamin C significantly increased by 4.4% for forced expiratory volume in 1 s (1.13 (0.02) vs. 1.09 (0.02) L; 0.05 (95% CI, 0.01–0.09); *p* = 0.02) Vitamin C supplementation improved forced expiratory flow at 25–75% of forced vital capacity by 18.4% (1.19 (0.03) vs. 1.001 (0.03); adjusted mean difference, 0.18 (95% CI, 0.10 to 0.26); *p* < 0.001) Supplemental vitamin C significantly decreased occurrence of wheezing between 4 to 6 years of age (28.3% vs. 47.2%; OR, 0.41 (95% CI, 0.23–0.74); *p* = 0.003)

* VCSIP = vitamin C to decrease the effects of smoking in pregnancy on infant lung function. * The study by McEvoy et al., 2014 [64] is a stand-alone cohort, and McEvoy et al., 2020 [66] and 2023 [67] are follow-up studies to the McEvoy et al., 2019 [65] VCSIP cohort.

## Data Availability

No new data were created. The full search strategy is found in the appendix, and any questions or concerns may be sent to the corresponding authors.

## Appendix A

Full Search Strategy:

(Prenatal Substance Use) AND (Maternal Nutrient Intakes OR Maternal Supplements OR Supplement Use OR Supplementation OR Vitamins and Minerals OR Antioxidants OR Iron OR Choline OR Maternal Nutrition OR Supplement Intake OR Maternal Supplements) AND (Fetal Outcomes OR Offspring Outcomes OR Cognitive Development Outcomes).
